# Knowledge, attitudes, and practices of pharmacy students and graduates regarding artificial intelligence in healthcare: a multi-regional cross-sectional study predominantly from the Asir region of Saudi Arabia

**DOI:** 10.3389/fpubh.2026.1736417

**Published:** 2026-02-18

**Authors:** Krishnaraju Venkatesan, Yahya I. Asiri, Pooja Muralidharan, Durgaramani Sivadasan, Hassabelrasoul Elfadil, Nouf M. Alharthi, Habab A. Osman, Sirajudeen Sheikh Alavudeen, Noohu Abdulla Khan, Vigneshwaran Easwaran, Premalatha Paulsamy, Kousalya Prabahar

**Affiliations:** 1 Department of Pharmacology, College of Pharmacy, King Khalid University, Abha, Aseer Province, Saudi Arabia; 2 PSG College of Pharmacy, Coimbatore, Tamilnadu, India; 3 Department of Pharmaceutics, College of Pharmacy, Jazan University, Jazan, Saudi Arabia; 4 Division of Microbiology, Immunology and Biotechnology, Department of Natural Products and Alternative Medicine, Faculty of Pharmacy, University of Tabuk, Tabuk, Saudi Arabia; 5 Department of Pharmacy, King Khalid University Medical City, Aseer region, Saudi Arabia; 6 Department of Education, Psychology and Mental Health, College of Education and Arts University of Tabuk, Tabuk, Saudi Arabia; 7 Department of Clinical Pharmacy, College of Pharmacy, King Khalid University, Abha, Saudi Arabia; 8 College of Nursing, Mahalah Branch for Girls King Khalid University, Khamis Mushayt, Saudi Arabia; 9 Department of Pharmacy Practice, Faculty of Pharmacy, University of Tabuk, Tabuk, Saudi Arabia

**Keywords:** Abha, artificial intelligence, knowledge, attitude, practice (KAP), pharmacy education, Saudi Arabia

## Abstract

**Background:**

Artificial intelligence (AI) is increasingly integrated into healthcare education and practice. However, evidence regarding AI utilization among pharmacy students and graduates in Saudi Arabia remains limited, particularly outside major urban centers. This study aimed to assess the knowledge, attitudes, and practices (KAP) related to AI among pharmacy students and graduates using a multi-regional sample predominantly from the Asir region.

**Methods:**

A cross-sectional, questionnaire-based survey was conducted among pharmacy students and graduates across multiple regions of Saudi Arabia. Demographic characteristics and KAP toward AI applications in pharmacy were collected using an electronic questionnaire. Of the 333 respondents, 200 (60.1%) were from the Asir region (including Abha and surrounding cities), while 133 (39.9%) represented other regions of Saudi Arabia. Participants included Diploma, B. Pharm, Pharm D, M. Pharm, and Ph.D. students and graduates.

**Results:**

Among the 333 respondents, most were aged 18–39 years and female. A high proportion (82.6%) reported awareness of AI, and 76.9% were familiar with ChatGPT. However, only 42.3% reported using AI tools for academic or professional purposes, revealing a significant awareness–practice gap (*p* < 0.05). Overall attitudes toward AI were positive, with 71.2% agreeing that AI can enhance pharmacy education and 68.4% believing it can improve clinical decision-making, although concerns regarding patient safety and workforce implications were commonly reported.

**Conclusions:**

In this multi-regional convenience sample, predominantly representing the Asir region (60.1%), pharmacy students and graduates demonstrated high awareness of artificial intelligence but limited practical utilization, indicating a persistent awareness–practice gap. Given the predominance of respondents from the Asir region, the regional findings should be interpreted as exploratory and are not nationally representative. Nevertheless, the results highlight the need for structured curricular integration of AI education, including formal AI literacy training, ethical guidance, and supervised exposure to AI-based tools, to promote consistent and responsible adoption across pharmacy education and practice in Saudi Arabia.

## 1 Introduction

Over the past five decades, artificial intelligence (AI) in healthcare has progressed substantially, driven by advances in machine learning (ML), data science (DS), and deep learning (DL). These developments have transformed diagnosis, clinical decision-making, prognostics, and rehabilitation, improving diagnostic precision, workflow efficiency, and patient outcomes (1–3). As AI becomes embedded in clinical workflows, healthcare professionals increasingly require not only clinical expertise but also foundational AI literacy. Pharmacists, who are central to medication therapy management, medication reconciliation, medication review, patient counseling, drug information services, adverse drug reaction (ADR) monitoring, and interprofessional collaboration (4, 5), are likely to use AI to analyse large datasets and support personalized care. AI tools therefore have the potential to enhance both pharmacy education and healthcare service delivery (3, 6), making AI competency an emerging expectation in pharmacy practice.

The integration of AI in education promotes creative learning skills and offers several advantages in medical and pharmacy education. In Saudi Arabia, the incorporation of AI into pharmacy training reflects a broader global shift toward digital transformation and competency-based training in healthcare (7, 8). These rapid advancements have generated both optimism and uncertainty among pharmacy learners regarding the evolving role of AI technologies (9). Pharmacy students and graduates are increasingly exposed to contemporary AI tools, including large language models such as ChatGPT, clinical decision-support systems, and AI-assisted medication management platforms, but the extent and quality of this exposure remain unclear. Despite progress in pharmacy education and expanding employment opportunities, a substantial knowledge gap concerning AI technologies persists, partly because AI capabilities are evolving faster than traditional curricula can adapt (3, 9).

Knowledge, Attitude and Practice (KAP) studies are useful for identifying such gaps and guiding AI-related training and curriculum development (10). In the Middle East, including Saudi Arabia, there is growing interest in the benefits and challenges of AI integration in healthcare education and practice (1, 7, 11, 12), yet pharmacy-specific KAP evidence remains limited and fragmented across institutions. This fragmented evidence base makes it difficult for educators and policymakers to form a coherent, pharmacy-focused view of AI readiness at the broader level.

Several peer-reviewed studies in Saudi Arabia have already examined AI-related perceptions among health-professional learners. Pharmacy undergraduates at King Saud University in Riyadh were surveyed regarding their perceptions of AI (13), and AI-related KAP has been evaluated among healthcare workers in private clinics in Jeddah (6). Broader investigations have assessed readiness for AI adoption among health-science students from multiple regions (14). However, these studies vary in scope, sample composition, and outcomes, and many focus on either awareness or attitudes alone, without comprehensively capturing pharmacy-specific AI readiness (4, 7, 14). In addition, most of the existing work concentrates solely on students, with limited information on pharmacy graduates who are transitioning into or already engaged in clinical practice. There is also comparatively little data that jointly examines knowledge, attitudes, practices, and perceived needs (KAP + Need) in relation to the newer generation of AI tools now entering pharmacy education and practice. Accordingly, the present study provides a unique contribution by offering the first comprehensive, multi-regional assessment of KAP + Need among both pharmacy students and pharmacy graduates in Saudi Arabia, while also evaluating familiarity with emerging AI tools such as clinical decision-support systems and large language models. This integrated approach addresses critical evidence gaps not captured in earlier Saudi-based research and enhances the broader relevance of the findings.

Students have also expressed concerns about how AI may affect future job opportunities, and these perceptions can influence AI acceptance and preparedness, making them highly relevant for curriculum reform and policy planning (15, 16). Recent literature from 2024–2025 highlights increasing use of AI-driven tools in clinical decision-making, patient communication, and health-professional education (4, 7, 17), underscoring the need to reassess current awareness, attitudes, and practices among pharmacy learners in light of rapidly changing technology. Within this evolving landscape, the central problem addressed by the present study is the lack of comprehensive, pharmacy-specific data on AI-related knowledge, attitudes, practices, and perceived needs among both pharmacy students and pharmacy graduates in Saudi Arabia. By providing updated multi-region data on KAP + Need and explicitly considering contemporary AI tools used in education and practice, this study aims to generate evidence that can inform curriculum development, faculty training, and policy initiatives for responsible AI integration in pharmacy education and practice.

## 2 Aim and objectives

This study aimed to assess the knowledge, attitudes, practices, and perceived needs (KAP + Need) related to artificial intelligence among pharmacy students and pharmacy graduates in Saudi Arabia. Specifically, we sought to: (i) describe levels of knowledge about AI concepts and applications in healthcare and pharmacy; (ii) characterize attitudes and perceptions toward AI in pharmacy education and practice; (iii) explore self-reported use of AI tools for educational and professional purposes; and (iv) identify perceived training needs and examine associations between KAP + Need and key sociodemographic or educational characteristics, recognizing that any regional comparisons are exploratory given the sample distribution.

## 3 Methods

### 3.1 Study design and tool

This observational, cross-sectional survey was conducted among pharmacy students and pharmacy graduates in Saudi Arabia using an online, self-administered questionnaire. The instrument was structured according to a Knowledge-Attitudes-Practice (KAP) framework and informed by constructs from technology-acceptance models (perceived usefulness, perceived ease of use, and behavioral intention) ensuring comprehensive assessment of both cognitive and behavioral dimensions of AI adoption in pharmacy education and practice. Item generation was based on a review of published surveys on AI and digital health in pharmacy and other health-professional education, as well as broader reports on digital transformation in Saudi healthcare. Existing KAP instruments on AI and digital health in pharmacy and other health professions were reviewed and adapted to guide the domain structure (KAP and practice) and the choice of response scales. In this study, items referring to AI “accuracy,” “reliability,” or “safety” were intentionally classified under the perception domain, as they reflect respondents' subjective beliefs and trust judgments rather than objectively verifiable factual knowledge.

The final questionnaire comprised four sections: (i) sociodemographic and educational characteristics (e.g., age, gender, region, current role, and highest pharmacy qualification); (ii) knowledge of AI concepts and applications in healthcare and pharmacy; (iii) attitudes and perceptions regarding the benefits, risks, and future role of AI in pharmacy education and practice; and (iv) self-reported practices and intended use of AI tools in educational and professional contexts. Knowledge items were single-best-answer questions focusing on fundamental definitions, examples of AI applications across the medication-use process, and awareness of specific AI platforms, including both widely discussed general-purpose tools and specialized healthcare AI systems (e.g., IBM Watson, PathAI), with response options such as “Yes/Correct,” “No/Incorrect,” or “Not sure.” These items were designed to capture recognition and conceptual understanding rather than hands-on experience with these tools. Many of the specialized healthcare AI systems included in the knowledge items require institutional licensing or integration into clinical environments and are therefore not routinely accessible to students or early-career practitioners, which informed the distinction between awareness and actual use. For analysis, correct answers were coded as 1 and incorrect/“Not sure” as 0, and individual items were summarized as proportions of correct responses; in addition, total knowledge scores were derived by summing item scores, with higher scores indicating better knowledge.

Attitudes and perceptions were assessed using five-point Likert-type items (strongly disagree to strongly agree) that captured agreement with statements about AI impact on efficiency, safety, professional roles, and ethical/data-governance issues; higher scores reflected more positive attitudes and greater perceived usefulness of AI. Practice items asked respondents to indicate how often they used AI tools (e.g., LLM-based chatbots, AI-enabled drug-information systems, or electronic prescribing decision support) for learning, research, or clinical tasks, using ordinal frequency options (never, rarely, sometimes, often, very often), with higher values indicating more frequent use. These items did not specify a fixed recall window (e.g., past month or past year) and were intended to capture respondents' typical or usual frequency of AI use, rather than usage within a predefined time period. The choice of Likert-type and ordinal frequency scales was based on their established use in KAP and technology-acceptance research and their alignment with previous AI-in-education surveys.

Attitudes and perceptions were assessed using five-point Likert-type items (strongly disagree to strongly agree) that captured agreement with statements regarding the impact of artificial intelligence on efficiency, safety, professional roles, and ethical or data-governance issues; higher scores reflected more positive attitudes and greater perceived usefulness of AI. Practice was assessed using categorical items that asked respondents to identify the artificial intelligence tools or software they had used for academic, research, or professional purposes (e.g., large language model-based chatbots, AI-enabled drug-information resources, or electronic prescribing and clinical decision-support systems). These items were designed to capture self-reported exposure and hands-on use of specific AI applications, rather than the frequency or intensity of use. As no fixed recall window (e.g., past month or past year) was specified, responses reflect participants' general experience with AI tools rather than usage within a predefined time frame. The choice of item formats was guided by their established use in KAP-based surveys and previous studies on AI adoption in health-professional education.

To further clarify construct boundaries, the perception domain was conceptualized to capture respondents' beliefs, trust, and subjective appraisals of AI tools, distinguishing it from both the knowledge and attitude domains. Knowledge items assessed factual understanding of AI concepts and applications, whereas attitude items reflected evaluative or affective judgments about AI integration in pharmacy education and practice. In contrast, perception items addressed respondents' interpretations of reliability, accuracy, safety, and potential risks, such as concerns about false information, data accuracy, or possible misuse. These items reflected belief-driven judgments rather than factual correctness, thereby justifying retention of perceptions as a separate analytical domain despite anticipated heterogeneity. These “accuracy”-related items were framed as belief-based evaluations of AI performance rather than assessments of respondents' factual correctness, thereby distinguishing perception from knowledge. Accordingly, accuracy-related items were retained within the perception domain rather than reclassified as knowledge items.

#### 3.1.1 Validation of the questionnaire

The questionnaire underwent a two-step validation process. First, content validity was evaluated by a panel of experts in pharmacy practice, clinical pharmacy, and pharmacy education, who independently reviewed all items for relevance, clarity, and coverage of key AI-related domains. Based on their feedback, items with overlapping content were merged or removed, and wording was refined to improve precision and readability. Second, the revised instrument was piloted among a convenience sample of 15 pharmacy students and recent pharmacy graduates to assess comprehensibility, survey flow, and completion time. Based on pilot feedback, minor wording changes were made (e.g., simplification of technical terms and clarification of AI-related examples), and some items were reordered to improve logical flow; no new domains were added and pilot data were not included in the final analysis. This process ensured that the instrument was conceptually grounded in the KAP and technology-acceptance frameworks while remaining understandable and context-appropriate for the target population.

Internal consistency reliability of the questionnaire domains was assessed using Cronbach's alpha coefficient. Reliability analyses were conducted separately for the knowledge, attitude, perception, and practice domains. A Cronbach's alpha value of ≥0.70 was considered indicative of acceptable internal consistency. Reliability testing was performed on the final study sample, as the pilot sample was small and intended only for face validity and comprehensibility assessment.

### 3.2 Study period and data collection and sampling strategy

Data collection was conducted between October 2024 and November 2024 using an online survey platform. The survey link was disseminated via institutional email lists, student and alumni groups, and professional social media channels, using an open invitation approach. Participants were also encouraged to share the survey link within their academic and professional networks to enhance participation from regions with initially low response rates. Because the survey was distributed through open electronic channels rather than a closed sampling frame, it was not possible to determine how many individuals actually received, viewed, or were eligible to participate in the survey. Consequently, a conventional response rate (i.e., the proportion of invited individuals who responded) could not be calculated. This approach may introduce selection bias, as participation was voluntary and may have preferentially attracted respondents with greater interest or familiarity with artificial intelligence. As a result, the findings should be interpreted with caution, particularly with respect to representativeness and generalizability beyond the surveyed population.

Participants were eligible for inclusion if they were (i) currently enrolled in a pharmacy programme (Diploma, B Pharm, M pharm or PharmD) or (ii) pharmacy graduates holding a Diploma, Bachelor's, Master's, or PhD degree, and (iii) residing in Saudi Arabia at the time of the survey. Participants were excluded if they did not provide informed consent or submitted incomplete questionnaires.

Because these channels did not allow tracking of how many individuals actually viewed or received the link, a conventional response rate could not be calculated. Further, to encourage multi-region participation, we collaborated with faculty contacts in multiple regions. Despite this, participation was disproportionately higher from the Asir region, and regional comparisons are therefore interpreted as exploratory. Accordingly, sampling was purely convenience-based rather than stratified by region.

The target sample size for the primary objective (estimation of overall KAP toward AI among pharmacy learners and graduates) was calculated using a single-proportion formula, assuming a conservative expected proportion, 95% confidence level, and 5% margin of error, yielding a minimum required sample of approximately 333 participants. No formal power calculation was performed for regional subgroup comparisons because the primary aim was overall KAP estimation in a multi-region convenience sample. Accordingly, any analyses by region are presented as exploratory and interpreted with caution, recognizing that some regions contributed relatively small numbers of respondents. As a result, the study is not powered to detect modest regional differences, and we have avoided making strong generalizations about regional effects. Participants were eligible if they were currently enrolled in a pharmacy programme or held a pharmacy degree (Diploma, BPharm, PharmD, Master's, or PhD) and were residing in Saudi Arabia at the time of the survey. Individuals who did not provide informed consent or submitted incomplete questionnaires were excluded.

### 3.3 Data analysis

Statistical analyses were performed using SPSS (version 24.0; IBM Corp., Armonk, NY, USA). Descriptive statistics (frequencies and percentages) were used to summarize sociodemographic characteristics and item-level responses for the KAP domains. For bivariate analyses, associations between KAP variables and key sociodemographic/educational factors were examined using chi-square tests or logistic regression, as appropriate. A *p*-value < 0.05 was considered statistically significant. Cramer's *V* values are reported alongside corresponding *p*-values in relevant tables.

Bivariate analyses were performed using logistic regression to examine associations between demographic variables and knowledge, attitude, and perception domains related to artificial intelligence. Results are presented as odds ratios (ORs) with 95% confidence intervals (CIs). For categorical variables with small cell counts, results were interpreted with caution due to the potential instability of estimates. Regional subgroup analyses were conducted as exploratory analyses because of the uneven geographic distribution of participants and the relatively small sample sizes within certain regions. No *post-hoc* category merging was performed unless conceptually justified, in order to preserve interpretability. Accordingly, findings from subgroup and regional analyses are intended to be hypothesis-generating rather than definitive.

The internal consistency of the questionnaire was assessed using Cronbach's alpha (α). Each domain of the KAP, Perception, and Need for AI was evaluated separately to determine the reliability of its items. Cronbach's alpha values were computed after data collection to examine how well the items within each scale measured their respective constructs. Alpha values ≥0.70 were considered acceptable, while values ≥0.80 indicated good reliability. This analysis ensured that the questionnaire items were coherent, consistent, and suitable for measuring the targeted domains within the study population.

### 3.4 Ethical considerations

This study involved human participants who voluntarily completed an online survey. Ethical approval was obtained from the King Khalid University Research Ethics Committee (HAPO-06-B-001; Approval No. ECM#2024-2805). The research adhered to the principles of the World Medical Association Declaration of Helsinki. All participants were informed about the study objectives, assured of confidentiality, and notified that no personally identifiable information would be collected. Participation was entirely voluntary, and individuals could withdraw from the survey at any time.

## 4 Results

### 4.1 Demographics

A total of 333 participants responded to the distributed questionnaire over a 2-month period. Table 1 shows the sociodemographic characteristics of participants. Of these, more than half participants were female 194 (58.3%). Among the study population, the majority of respondents were from the Asir region (21.9%, *n* = 73), followed by Najran (12.3%, *n* = 41), Khamis Mushayt (16.8%, *n* = 56), and Al Madinah Al Munawwarah (3.6%, *n* = 12). Al Madinah Al Munawwarah (2.7%, *n* = 9). This correction was made to ensure the description accurately reflects the data presented in Table 1. Most participants (98.2%) were classified as youth (18–39 years). Almost half of the respondents were students pursuing a pharmacy degree (48.0%, *n* = 160), followed by B. Pharmacy graduates (46.3%, *n* = 157). A smaller number were enrolled in M. Pharmacy (2.7%, *n* = 9) and Ph.D. programs (1.5%, *n* = 5). Regarding occupational distribution, the majority were students (55.9%, *n* = 186), followed by participants employed in the private sector (21.0%, *n* = 70).

**Table 1:** Sociodemographic characteristics of study participants (*n* = 333).

**Variables**	***n***	**Percentage**
**Sex**		
Male	139	41.7%
Female	194	58.3%
**Age in years**		
Youth (18–39)	327	98.2%
Middle-aged (40–59)	6	1.8%
**Place of residence**		
**Asir region**		
Abha	73	21.9%
Barq	25	7.5%
Bisha	8	2.4%
Khamis Mushayt	56	16.8%
Mahayil	5	1.5%
Sarat Ubaida	8	2.4%
Tharban	15	4.5%
The Majardah	10	3.0%
**Non-Asir region**		
AL Madinah AL Munawwarah	9	2.7%
Al-Qassim	12	3.6%
Hail	9	2.7%
Jazan	12	3.6%
Mecca	20	6.0%
Najran	6	1.8%
Riyadh	41	12.3%
Tabuk	13	3.9%
Taif	11	3.3%
**Education**		
Pharmacy students	160	48.0%
Pharmacy graduates – diploma	2	6.0%
Pharmacy graduates – bachelor's	157	46.3%
Pharmacy graduates – master's	9	2.7%
Pharmacy graduates – Ph. D	5	1.5%
**Occupation**		
Not employed	52	15.6%
Students	186	55.9%
Business	7	2.1%
Private	70	21.0%
Government	18	5.4%

A notable regional skew was observed in the sample composition. Approximately 60% of the respondents were from the Asir region when all cities within Asir including Abha, Khamis Mushayt, Barq, Tharban, Bisha, Mahayil, Sarat Ubaida and The Majardah. The remaining 40% of participants represented other regions across Saudi Arabia (non-Asir region). This distribution reflects the higher accessibility and engagement of participants from the Asir region, which may influence the representativeness of regional perceptions toward AI. However, clarifying this distribution provides transparency and ensures accurate interpretation of the findings.

The results showed that a strong majority of participants demonstrated awareness of pharmacy-related AI tools, including clinical decision-support systems, AI-based medication management platforms, and commonly used large language models.

### 4.2 Pharmacist's knowledge on AI

Table 2 illustrates participants' knowledge of AI across different pharmacy education levels. A majority of pharmacy students (47.4%) and bachelor's pharmacists (44.4%) reported having heard about AI programs, indicating a strong general awareness (*p* = 0.6, Cramer's *V* = 0.091). Most respondents acknowledged AI as a useful tool for medication management (33 and 35.4%; *p* = 0.1, Cramer's *V* = 0.133), patient safety (34.2 and 36.0%; *p* = 0.2, Cramer's *V* = 0.128), and cancer research (28.5 and 33.9%; *p* = 0.2, Cramer's *V* = 0.120). Participants also recognized AI's utility in clinical trials (*p* = 0.8, Cramer's *V* = 0.080), drug discovery (*p* = 0.2, Cramer's *V* = 0.121), personalized treatment (*p* = 0.4, Cramer's *V* = 0.107), and pharmacy supply chain management including clinical decision-support systems (χ^2^ = 0.6, *p* = 0.097, Cramer's *V* = 0.097). Only a few respondents expressed uncertainty or lack of knowledge. Overall, the results demonstrate that a strong majority of participants were aware of pharmacy-related AI tools, including clinical decision-support systems, AI-based medication management platforms, and commonly used large language models.

**Table 2:** Participants knowledge on AI programs with education qualification (*n* = 333).

**Variables**		***n*** **(%)**					**χ^2^ “*p*”*-*Value (Cramer's “*V*”)**
		**Pharmacy student**	**Pharmacist–diploma**	**Pharmacist–bachelor's**	**Pharmacist–master's**	**Pharmacist–Ph. D**	
Have you heard about AI programs?	Yes	158 (47.4%)	2 (0.6%)	148 (44.4%)	9 (2.7%)	5 (1.5%)	0.6 (0.091)
	No	1 (0.3%)	0 (0%)	5 (1.5%)	0 (0%)	0 (0%)	
	I'm not sure	1 (0.3%)	0 (0%)	4 (1.2%)	0 (0%)	0 (0%)	
Is AI useful for better medication management?	Yes	110 (33%)	2 (0.6%)	118 (35.4%)	9 (2.7%)	2 (0.6%)	0.1 (0.133)
	No	16 (4.8%)	0 (0%)	14 (4.2%)	0 (0%)	0 (0%)	
	I'm not sure	34 (10.2%)	0 (0%)	25 (7.5%)	0 (0%)	3 (0.9 %)	
Is AI useful for improved patient safety and outcomes	Yes	114 (34.2%)	2 (0.6%)	120 (36.0%)	9 (2.7%)	2 (0.6%)	0.2 (0.128)
	No	14 (4.2%)	0 (0%)	11 (3.3%)	0 (0%)	0 (0%)	
	I'm not sure	32 (9.6%)	0 (0%)	26 (7.8%)	0 (0.0%)	3 (0.9%)	
Is AI useful for cancer research?	Yes	95 (28.5%)	2 (0.6%)	113 (33.9%)	8 (2.4%)	3 (0.9%)	0.2 (0.120)
	No	14 (4.2%)	0 (0%)	10 (3.0%)	0 (0%)	0 (0%)	
	I'm not sure	51 (15.3%)	0 (0%)	34 (10.2%)	1 (0.3%)	2 (0.6%)	
Is AI useful for clinical trials?	Yes	99 (29.7%)	2 (0.6%)	100 (30.0%)	8 (2.4%)	3 (0.9%)	0.8 (0.080)
	No	25 (7.5%)	0 (0%)	25 (7.5%)	0 (0%)	1 (0.3%)	
	I'm not sure	36 (10.8%)	0 (0%)	32 (9.6%)	1 (0.3%)	1 (0.3%)	
Is AI useful for drug discovery?	Yes	98 (29.4%)	2 (0.6%)	98 (29.4%)	9 (2.7%)	3 (0.9%)	0.2 (0.121)
	No	28 (8.4%)	0 (0%)	20 (6.0%)	0 (0%)	0 (0%)	
	I'm not sure	34 (10.2%)	0 (0%)	39 (11.7%)	0 (0%)	2 (0.6%)	
Is AI useful for personalized treatment?	Yes	94 (28.2%)	1 (0.3%)	87 (26.1%)	5 (1.5%)	5 (1.5%)	0.4 (0.107)
	No	27 (8.1%)	1 (0.3%)	30 (9%)	3 (0.9%)	0 (0%)	
	I'm not sure	39 (11.7%)	0 (0%)	40 (12.0%)	1 (0.3%)	0 (0%)	
Is AI useful for pharmacy supply chain management and clinical decision support system (CDSS)	Yes	109 (32.7%)	2 (0.6%)	111 (33.3%)	7 (2.1%)	3 (0.9%)	0.6 (0.097)
	No	16 (4.8%)	0 (0%)	13 (3.9%)	2 (0.6%)	0 (0%)	
	I'm not sure	35 (10.5%)	0 (0%)	33 (9.9%)	0 (0%)	2 (0.6%)	

^*^p < 0.05—significant, χ^2^- Chi-square.

Cells with counts < 5 are indicated in the table; interpret chi-square results with caution.

Chi-square test may not be fully reliable due to small expected counts in several categories; Cramer's V is reported to indicate effect size.

### 4.3 Pharmacist's attitude on AI programs

Table 3 presents the attitudes of pharmacy students and professionals toward the application of AI in pharmacy practice. A majority of respondents, particularly pharmacy students (36.0%) and bachelor's degree pharmacists (37.5%), showed a positive attitude toward the usefulness of AI in identifying medication errors, high-risk drug dosing, and drug–drug interactions (*p* = 0.08, Cramer's *V* = 0.145). Around 27.3% of students and 23.4% of bachelor pharmacists agreed that AI- and ML-based systems can support personalized dosage recommendations (*p* = 0.2, Cramer's *V* = 0.124). Regarding emerging technologies, attitudes toward the usefulness of NFC tags in pharmacy practice were mixed, with 29.7% of students and 18.0% of bachelor pharmacists expressing agreement, while a larger proportion remained skeptical or disagreed (*p* = 0.9, Cramer's *V* = 0.034).

**Table 3:** Participants attitude on AI programs with education qualification (*n* = 333).

**Variables**		***n*** **(%)**					**χ^2^ “*p*”*-*Value (Cramer's “*V*”)**
		**Pharmacy student**	**Pharmacist–diploma**	**Pharmacist–bachelor's**	**Pharmacist–master's**	**Pharmacist–Ph. D**	
Is AI useful for identification of medication errors like high-risk drug dosing and drug-drug interactions?	Yes	120 (36.0%)	2 (0.6%)	125 (37.5%)	7 (2.1%)	2 (0.6%)	0.08 (0.145)
	No	18 (5.4%)	0 (0%)	13 (3.9%)	2 (0.6%)	0 (0%)	
	I'm not sure	22 (6.6%)	0 (0%)	19 (5.7%)	0 (0%)	3 (0.9%)	
Patients can benefit from a personalized AI/ML-based dosage recommendation system	I agree	91 (27.3%)	1 (0.3%)	78 (23.4%)	7 (2.1%)	4 (1.2%)	0.2 (0.124)
	I disagree	69 (20.7%)	1 (0.3%)	79 (23.7%)	2 (0.6%)	1 (0.3%)	
AI could aid in drug selection decisions by indicating which patients are unlikely to experience adverse effects from a specific drug	I agree	95 (28.5%)	1 (0.3%)	88 (26.4%)	5 (1.5%)	4 (1.2%)	0.8 (0.066)
	I disagree	65 (19.5%)	1 (0.3%)	69 (20.7%)	4 (1.2%)	1 (0.3%)	
AL/ML (machine learning) algorithms are increasingly being used to develop predictive models for Potentially inappropriate medications (PIMs) prescription	I agree	84 (25.2%)	1 (0.3%)	86 (25.2%)	6 (1.8%)	3 (0.9%)	0.9 (0.051)
	I disagree	76 (22.8%)	1 (0.3%)	71 (21.3%)	3 (0.9%)	2 (0.6%)	
NFC tags would be useful in pharmacy practice	I agree	59 (29.7%)	1 (0.3%)	60 (18.0%)	4 (1.2%)	2 (0.6%)	0.9 (0.034)
	I disagree	101 (17.7%)	1 (0.3%)	97 (29.1%)	5 (1.5%)	3 (0.9%)	
AI assists in advancing public health monitoring by analyzing large-scale health data sets to detect trends in disease outbreaks, drug usage patterns, and other public health issues that may require a response by pharmacists or public health organizations	I agree	115 (34.5%)	2 (0.6%)	120 (36%)	8 (2.4%)	4 (1.2%)	0.6 (0.089)
	I disagree	45 (13.5%)	0 (0%)	37 (11.1%)	1 (0.3%)	1 (0.3%)	
Secondly, AI can improve automated dispensing systems (ADSs) by increasing accuracy and precision in dispensing, learn from past errors, and employ machine learning algorithms for continuous system optimization	I agree	107 (32.1%)	2 (0.6%)	115 (34.5%)	9 (2.7%)	4 (1.2%)	0.1 (0.139)
	I disagree	53 (15.9%)	0 (0%)	42 (12.6%)	0 (0%)	1 (0.3%)	
AI enhances supply chain management. by analyzing a vast amount of data, including past sales, seasonality, local health trends, promotional activities, and even external factors like weather patterns or disease outbreaks, to predict demand for various medications	I agree	111 (33.3%)	2 (0.6%)	128 (38.4%)	9 (2.7%)	5 (2.0%)	0.02^*^ (0.187)
	I disagree	49 (14.7%)	0 (0%)	29 (8.7%)	0 (0%)	0 (0%)	
AI tools can boost pharmacies profitability by analyzing data such as past sales and local health trends, AI algorithms can predict medication demand	I agree	123 (36.9%)	2 (0.6%)	129 (38.7%)	9 (2.7%)	5 (2.0%)	0.2 (0.128)
	I disagree	37 (11.1%)	0 (0%)	28 (8.4%)	0 (0%)	0 (0%)	
IBM Watson for Oncology is an AI-driven tool that aids oncologists in pinpointing potential cancer drug treatments tailored to individual patient profiles	I agree	83 (24.9%)	2 (0.6%)	88 (26.4%)	8 (2.4%)	2 (0.6%)	0.1 (0.145)
	I disagree	77 (23.1%)	0 (0%)	69 (20.7%)	1 (0.3%)	3 (0.9%)	
Tools like Google DeepMind have shown the ability to accurately forecast hospital readmission rates for heart failure patients, providing pharmacists with a more precise tool for customizing care strategies	I agree	85 (25.5%)	2 (0.6%)	80 (24.0%)	8 (2.4%)	2 (0.6%)	0.1 (0.145)
	I disagree	75 (22.5%)	0 (0%)	77 (23.1%)	1 (0.3%)	3 (0.9%)	
Interactive chatbots, such as Buoy Health Citation, offer personalized advice about medication usage, diet changes, lifestyle modifications, and treatment plans, fostering increased understanding and compliance	I agree	94 (28.2%)	2 (0.6%)	98 (29.4%)	6 (1.8%)	3 (0.9%)	0.7 (0.075)
	I disagree	66 (19.8%)	0 (0%)	59 (17.7%)	3 (0.9%)	2 (0.6%)	
Platforms like Dosis personalized dosing use real-time patient data to suggest optimal medication dosages based on individual responses for better therapeutic outcomes	I agree	87 (26.1%)	2 (0.6%)	93 (27.9%)	7 (2.1%)	3 (0.9%)	0.4 (0.107)
	I disagree	73 (21.9%)	0 (0%)	64 (19.2%)	2 (0.6%)	2 (0.6%)	
Tools like PathAI, rapidly generate evidence-based treatment recommendations	I agree	82 (24.6%)	2 (0.6%)	79 (23.7%)	6 (1.8%)	2 (0.6%)	0.5 (0.096)
	I disagree	78 (23.4%)	0 (0%)	78 (23.4%)	3 (0.9%)	3 (0.9%)	

^*^p < 0.05—significant, χ^2^- Chi-square.

Cells with counts < 5 are indicated in the table; interpret chi-square results with caution.

Chi-square test may not be fully reliable due to small expected counts in several categories; Cramer's V is reported to indicate effect size.

A higher proportion of participants (34.5% students and 36.0% bachelor pharmacists) strongly agreed that AI supports public health monitoring and detection of disease trends (*p* = 0.6, Cramer's *V* = 0.089). Additionally, 33.3% of students and 38.4% of bachelor pharmacists recognized AI's role in optimizing supply chain management, and this association was statistically significant (*p* = 0.02, Cramer's *V* = 0.187). Furthermore, 28.2% of students and 29.4% of bachelor pharmacists acknowledged the usefulness of interactive chatbots like Buoy Health for promoting treatment adherence (*p* = 0.7, Cramer's *V* = 0.075). Overall, the findings indicate a generally positive attitude toward AI adoption in pharmacy operations, with bachelor's degree pharmacists and students showing the most supportive responses across all parameters.

It should be noted that Table 3 assesses respondents' conceptual awareness and perceived usefulness of various AI platforms used in healthcare and pharmacy practice, many of which (e.g., IBM Watson for Oncology, PathAI, and DeepMind applications) are institutionally deployed tools that typically require organizational licensing and are not intended for routine personal use. In contrast, Table 5 captures self-reported personal use of readily accessible AI tools, such as ChatGPT or Siri. This distinction explains the apparent discrepancy between high awareness or perceived usefulness of specialized AI systems and their limited reported personal use.

Collectively, these findings indicate broad acceptance of AI applications in pharmacy practice, with limited variation across educational levels and strongest support observed for supply chain optimization.

### 4.4 Pharmacist's practice on AI programs

The study evaluated self-reported experience with AI programs among pharmacy students and pharmacists across different qualifications (Table 4). Practice items captured whether respondents had ever used specific AI applications for academic, research, or professional purposes, rather than use within a predefined time frame (e.g., past month or year). The study evaluated the use of AI programs among pharmacy students and pharmacists across different qualifications (Table 4). Overall, a majority of participants reported using AI programs, with pharmacy students leading at 44.7%, followed by pharmacists with bachelor's degrees (36.3%), while usage was lower among diploma, master's, and Ph.D. holders (*p* < 0.001, Cramer's *V* = 0.238). When specifically asked about using AI for academic purposes, 43.5% of students and 35.4% of bachelor's pharmacists reported engagement, showing a statistically significant difference (*p* = 0.002, Cramer's *V* = 0.229). Diploma, master's, and Ph.D. pharmacists consistently reported lower usage across all AI applications, including retrieving drug information, exam preparation, performing medication-related duties, and writing research articles (*p*-values ranged from 0.01–0.1, Cramer's *V* = 0.142–0.193). These findings highlight that younger or academically enrolled participants are more likely to integrate AI into their studies and professional tasks. Overall, the trend suggests increasing AI adoption among pharmacy professionals, particularly for educational and research purposes.

**Table 4:** Participants practice on AI programs with education qualification (*n* = 333).

**Variables**		***n*** **(%)**					**χ^2^ “*p*”*-*Value (Cramer's “*V*”)**
		**Pharmacy student**	**Pharmacist–diploma**	**Pharmacist–bachelor's**	**Pharmacist–master's**	**Pharmacist–Ph. D**	
Have you used AI programs?	Yes	149 (44.7%)	2 (0.6%)	121 (36.3%)	8 (2.4%)	3 (0.9%)	< 0.001^**^ (0.238)
	No	11 (3.3%)	0 (0%)	36 (10.8%)	1 (0.3%)	2 (0.6%)	
Have you used AI for study?	Yes	145 (43.5%)	1 (0.3%)	118 (35.4%)	6 (1.8%)	3 (0.9%)	0.002^**^ (0.229)
	No	15 (4.5%)	1 (0.3%)	39 (11.7%)	3 (0.9%)	2 (0.6%)	
Have you used AI for getting drug information?	Yes	118 (35.4%)	1 (0.3%)	99 (29.7%)	7 (2.1%)	2 (0.6%)	0.1 (0.142)
	No	42 (12.6%)	1 (0.3%)	58 (17.4%)	2 (0.6%)	3 (0.9%)	
Do you use AI to find exam questions about drugs?	Yes	112 (36.6%)	1 (0.3%)	93 (27.9%)	6 (1.8%)	2 (0.6%)	0.01^*^ (0.193)
	No	38 (11.4%)	1 (0.3%)	64 (19.2%)	3 (0.9%)	3 (0.9%)	
Do you use AI to perform medication-related duties?	Yes	111 (33.3%)	1 (0.3%)	86 (25.8%)	6 (1.8%)	2 (0.6%)	0.07 (0.159)
	No	49 (14.7%)	1 (0.3%)	71 (21.3%)	3 (0.9%)	3 (0.9%)	
Have you used AI in writing research articles?	Yes	118 (35.4%)	2 (0.6%)	94 (28.2%)	7 (2.1%)	2 (0.6%)	0.04^*^ (0.173)
	No	42 (12.6%)	0 (0%)	63 (18.9%)	2 (0.6%)	3 (0.9%)	

^*^p < 0.05; ^**^p < 0.001—significant, χ^2^- Chi-square.

Cells with counts < 5 are indicated in the table; interpret chi-square results with caution.

Chi-square test may not be fully reliable due to small expected counts in several categories; Cramer's V is reported to indicate effect size.

Practice items reflect self-reported ever use or general experience with AI programs. No fixed recall period (e.g., past month or past year) was specified.

### 4.5 AI tools utilized by pharmacy professionals

Among the participants, ChatGPT was the most frequently used AI program, with 42.3% of pharmacy students and 36.0% of pharmacists with a bachelor's degree reporting usage (*p* < 0.001, Cramer's *V* = 0.244). Siri was used by 9.0% of students and 7.2% of bachelor's pharmacists (*p* = 0.01, Cramer's *V* = 0.195). Other AI tools, including Clalit AI, Drawn AI, Google Assistant, Pharmee, Robotic App, and miscellaneous programs, were used by a small fraction of participants (0.3%−4.8%) with no statistically significant differences between groups (*p* > 0.05, Cramer's *V* = 0.067–0.142). A large proportion of respondents reported not using AI programs, highlighting limited adoption across pharmacy professionals. Overall, ChatGPT appears to be the most preferred AI tool among participants, particularly among students and bachelor's degree holders (Table 5).

**Table 5:** Usage of AI programs among pharmacy professionals.

**AI program used**		***n*** **(%)**					**χ^2^ “*p*”*-*Value (Cramer's “*V*”)**
		**Pharmacy student**	**Pharmacist–diploma**	**Pharmacist–bachelor's**	**Pharmacist–master's**	**Pharmacist–Ph. D**	
Clalit AI	Used	15 (4.5%)	2 (0.6%)	13 (3.9%)	2 (0.6%)	0 (0%)	0.6 (0.091)
	Not used	145 (43.5%)	0 (0%)	144 (43.2%)	7 (2.1%)	5 (1.5%)	
Drawn AI	Used	8 (2.4%)	0 (0%)	6 (1.8%)	2 (0.6%)	0 (0%)	0.1 (0.142)
	Not used	152 (45.6%)	2 (0.6%)	151 (45.3 %)	7 (2.1%)	5 (1.5%)	
Google assistant	Used	27 (8.1%)	1 (0.3%)	36 (10.8%)	4 (1.2%)	1 (0.3%)	0.1 (0.135)
	Not used	133 (39.9%)	1 (0.3%)	121 (36.3%)	5 (1.5%)	4 (1.2%)	
ChatGPT	Used	141 (42.3%)	0 (0%)	120 (36.0%)	5 (1.5%)	4 (1.2%)	< 0.001^**^ (0.244)
	Not used	19 (5.7%)	2 (0.6%)	37 (11.1%)	4 (1.2%)	1 (0.3%)	
Pharmee	Used	16 (4.8%)	0 (0%)	14 (4.2%)	2 (0.6%)	1 (0.3%)	0.6 (0.087)
	Not used	144 (43.2%)	2 (0.6%)	143 (42.9%)	7 (2.1%)	4 (1.2%)	
Robotic App	Used	7 (2.1%)	0 (0%)	3 (0.9%)	0 (0%)	0 (0%)	0.7 (0.081)
	Not used	153 (45.9%)	2 (0.6%)	154 (46.2%)	9 (2.7%)	5 (1.5%)	
Siri	Used	30 (9.0%)	2 (0.6%)	24 (7.2%)	3 (0.9%)	0 (0%)	0.01^*^ (0.195)
	Not used	130 (39.0%)	0 (0%)	133 (39.9%)	6 (1.8%)	5 (1.5%)	
None	Used	16 (4.8%)	0 (0%)	22 (6.6%)	0 (0%)	1 (0.3%)	0.5 (0.096)
	Not used	144 (43.2%)	2 (0.6%)	135 (40.5%)	9 (2.7%)	4 (1.4%)	
Others	Used	13 (3.9%)	0 (0%)	9 (2.7%)	1 (0.3%)	0 (0%)	0.8 (0.067)
	Not used	147 (44.1%)	2 (0.6%)	148 (44.4%)	8 (2.4%)	5 (1.5%)	

^*^p < 0.05; ^**^p < 0.001—significant, χ^2^- Chi-square.

Cells with counts < 5 are indicated in the table; interpret chi-square results with caution.

Chi-square test may not be fully reliable due to small expected counts in several categories; Cramer's V is reported to indicate effect size.

Reported AI tools indicate general or prior use, not time-bound frequency.

### 4.6 Pharmacist perceptions of AI programs

Table 6 summarizes participants' perceptions and concerns regarding the use of AI programs in pharmacy education and practice. In this context, “perception” reflects respondents' subjective judgments, confidence, and concerns regarding AI-generated outputs rather than objective verification of AI performance. Items addressing data accuracy were interpreted as perceived reliability rather than factual knowledge. Approximately 12.3% of students and 12.9% of bachelor pharmacists perceived AI-generated drug data to be accurate, whereas 19.5% of students and 14.7% of bachelor pharmacists expressed skepticism regarding data accuracy (*p* = 0.07, Cramer's *V* = 0.147). A substantial proportion of respondents (36.0% of students and 33.6% of bachelor pharmacists) perceived that AI-based data collection may contain false information (*p* = 0.9, Cramer's *V* = 0.068).

**Table 6:** Perceptions and concerns about AI programs.

**Variables**		***n*** **(%)**					**χ^2^ “*p*”*-*Value (Cramer's “*V*”)**
		**Pharmacy student**	**Pharmacist–diploma**	**Pharmacist–bachelor's**	**Pharmacist–master's**	**Pharmacist–Ph. D**	
Are AI data on drugs accurate?	Yes	41 (12.3%)	1 (0.3%)	43 (12.9%)	6 (1.8%)	2 (0.6%)	0.07 (0.147)
	No	65 (19.5%)	0 (0%)	49 (14.7%)	1 (0.3%)	0 (0%)	
	I'm not sure	54 (16.2%)	1 (0.3%)	65 (19.5%)	2 (0.6%)	3 (0.9%)	
Might data collection using AI programs contain false information?	Yes	120 (36.0%)	1 (0.3%)	112 (33.6%)	7 (2.1%)	3 (0.9%)	0.9 (0.068)
	No	17 (5.1%)	0 (0%)	18 (5.4%)	1 (0.3%)	1 (0.3%)	
	I'm not sure	23 (6.9%)	1 (0.3%)	27 (8.1%)	1 (0.3%)	1 (0.3 %)	
Are AI programs helpful for student education?	Yes	141 (42.3%)	2 (0.6%)	129 (38.7%)	7 (2.1%)	4 (1.2%)	0.3 (0.118)
	No	7 (2.1%)	0 (0%)	9 (2.7%)	2 (0.6%)	0 (0%)	
	I'm not sure	12 (3.6%)	0 (0%)	19 (5.7%)	0 (0.0%)	1 (0.3%)	
Does AI programs help students cheat?	Yes	81 (24.3%)	2 (0.6%)	93 (27.9%)	8 (2.4%)	3 (0.9%)	0.3 (0.117)
	No	44 (13.2%)	0 (0%)	31 (9.3%)	0 (0%)	1 (0.3%)	
	I'm not sure	35 (10.5%)	0 (0%)	33 (9.9%)	1 (0.3%)	1 (0.3%)	
Are AI programs helpful for students understanding and motivation?	Yes	132 (39.6%)	2 (0.6%)	118 (35.4%)	7 (2.1%)	5 (1.5%)	0.8 (0.083)
	No	12 (3.6%)	0 (0%)	18 (5.4%)	1 (0.3%)	0 (0%)	
	I'm not sure	16 (4.8%)	0 (0%)	21 (6.3%)	1 (0.3%)	0 (0%)	

^*^p < 0.05—significant, χ^2^- Chi-square.

Cells with counts < 5 are indicated in the table; interpret chi-square results with caution.

Chi-square test may not be fully reliable due to small expected counts in several categories; Cramer's V is reported to indicate effect size.

Despite these concerns, attitudes toward the educational value of AI were predominantly positive, with 42.3% of students and 38.7% of bachelor pharmacists agreeing that AI programs are helpful for learning (*p* = 0.3, Cramer's *V* = 0.118). However, approximately one-quarter of respondents perceived that AI tools could facilitate academic dishonesty (*p* = 0.3, Cramer's *V* = 0.117). Overall, participants perceived AI programs as beneficial for enhancing understanding and motivation (*p* = 0.8, Cramer's *V* = 0.083), while simultaneously expressing concerns related to data reliability and potential misuse.

### 4.7 Need for AI in healthcare and research

Table 7 presents respondents' views on the perceived need for integrating AI into pharmacy education, healthcare practice, and research. This variable assesses participants' interest in incorporating AI-related topics into pharmacy curricula, adopting AI-enabled tools in clinical settings, and expanding their own knowledge of AI applications in healthcare and research. A substantial proportion of pharmacy students (37.2%) and bachelor pharmacists (39.0%) expressed interest in including AI-related content in the pharmacy curriculum (*p* = 0.4, Cramer's *V* = 0.104). Similarly, 38.4% of students and 39.6% of bachelor pharmacists supported implementing AI-enabled tools in community pharmacies and hospitals to improve patient care (*p* = 0.6, Cramer's *V* = 0.090). Interest in learning more about AI applications in both hospital and community settings was also high, with 41.1% of students and 41.7% of bachelor pharmacists responding positively (*p* = 0.7, Cramer's *V* = 0.071). Overall, these findings reflect a strong perceived need for integrating AI into pharmacy education, healthcare practice, and research domains, underscoring growing recognition of its potential to enhance efficiency, accuracy, and patient outcomes.

**Table 7:** The growing importance of AI in in healthcare and research with education qualification (*n* = 333).

**Variables**		***n*** **(%)**					**χ^2^ “*p*”*-*Value (Cramer's “*V*”)**
		**Pharmacy student**	**Pharmacist–diploma**	**Pharmacist–bachelor's**	**Pharmacist–master's**	**Pharmacist–Ph. D**	
Do you want to include information about artificial intelligence in pharmacy curricula?	Yes	124 (37.2%)	2 (0.6%)	130 (39%)	8 (2.4%)	5 (1.5%)	0.4 (0.104)
	No	36 (10.8%)	0 (0%)	27 (8.1%)	1 (0.3%)	0 (0%)	
Do you want to implement AI enabled tools in community pharmacies and hospitals to benefit the patient?	Yes	128 (38.4%)	2 (0.6%)	132 (39.6%)	8 (2.4%)	5 (1.5%)	0.6 (0.090)
	No	32 (9.6%)	0 (0%)	25 (7.5%)	1 (0.3%)	0 (0%)	
Want to learn more about AI in hospital and community settings?	Yes	137 (41.1%)	2 (0.6%)	139 (41.7%)	8 (2.4%)	5 (1.5%)	0.7 (0.071)
	No	23 (6.9%)	0 (0%)	18 (5.4%)	1 (0.3%)	0 (0%)	

^*^p < 0.05—significant, χ^2^- Chi-square.

Cells with counts < 5 are indicated in the table; interpret chi-square results with caution.

Chi-square test may not be fully reliable due to small expected counts in several categories; Cramer's V is reported to indicate effect size.

### 4.8 Reliability analysis of the questionnaire scales

The internal consistency of the scales used in this study was assessed using Cronbach's alpha (α). The Knowledge scale, comprising eight items, demonstrated good reliability with an α value of 0.798. The Attitude scale, which included 14 items, showed excellent internal consistency (α = 0.884). The Practice scale (six items) also exhibited good reliability (α = 0.804). The Perception scale, consisting of five items, yielded a moderate reliability coefficient (α = 0.592), indicating that some items may require refinement to enhance internal consistency. Lastly, the three-item Need for AI scale demonstrated acceptable reliability with an α value of 0.783. Overall, the majority of the scales showed good to excellent reliability, supporting their suitability for use in the study (Table 8).

**Table 8:** Reliability analysis (Cronbach's alpha) for KAP.

**Scale**	**Number of items**	**Cronbach's alpha (α)**
Knowledge	8	0.798
Attitude	14	0.884
Practice	6	0.804
Perception	5	0.592
Need for AI	3	0.783

### 4.9 Bivariate analysis of KAP toward AI in pharmacy practice

Before interpreting the regional comparisons presented in Table 9, it is important to note the uneven geographic distribution of the study sample. Of the 333 respondents, 200 participants (60.1%) were from the Asir region, while 133 participants (39.9%) represented all other regions combined. Consequently, all regional comparisons were conducted as exploratory analyses. These findings are not nationally representative and should be interpreted with caution, as they are intended to be hypothesis-generating rather than definitive assessments of regional differences in knowledge, attitudes, and perceptions toward artificial intelligence.

**Table 9:** Bivariate logistic regression of demographic factors with knowledge, attitude, and perception domains.

**Sociodemographic characteristics**	**Knowledge (good)**		**Attitude (positive and)**		**Perception (positive and)**	
	**and poor knowledge)**		**negative attitude)**		**negative perception)**	
	**OR (95% CI)**	**“** ***p*** **”-Value**	**OR (95% CI)**	**“** ***p*** **”-Value**	**OR (95% CI)**	**“** ***p*** **”-Value**
**Sex**						
Male	0.812 (0.519–1.270)	0.3	1.192 (0.709–2.004)	0.5	0.871 (0.562–1.348)	0.5
Female	Reference					
**Age**						
Youth (18–39)	0.808 (0.146–4.476)	0.8	0.659 (0.076–5.732)	0.7	0.569 (0.103–3.152)	0.5
Middle-aged (40–59)	Reference					
**Education qualification**						
Pharmacy students	1.880 (0.054–1.207)	0.4	1.556 (0.252–9.609)	0.6	1.743 (0.284–10.716)	0.5
Pharmacist diploma	0.00 (0.00–0.00)	0.9	0.00 (0.00–0.00)	0.9	1.500 (0.055–40.633)	0.8
Pharmacist—bachelor's	3.02 (0.491–18.69)	0.2	3.071 (0.490–19.248)	0.2	1.726 (0.281–10.615)	0.5
Pharmacist—master's	12.00 (0.773–186.36)	0.07	0.00 (0.00–0.00)	0.9	3.000 (0.312–28.841)	0.3
Pharmacist—Ph. D	Reference					
**Occupation**						
Not employed	0.524 (0.163–1.688)	0.2	0.417 (0.084–2.075)	0.2	0.634 (0.216–1.866)	0.4
Students	0.521 (0.178–1.521)	0.2	0.314 (0.070–1.412)	0.1	1.037 (0.392–2.764)	0.9
Business	0.513 (0.083–3.158)	0.4	0.750 (0.057–9.871)	0.8	0.600 (0.103–3.495)	0.5
Private	1.111 (0.347–3.553)	0.8	0.750 (0.149–3.772)	0.7	0.950 (0.335–2.695)	0.9
Government	Reference					
**Place of residence**						
**Asir region**						
Abha	1.455 (0.407–5.196)	0.5	2.969 (0.801–11.002)	0.1	5.455 (1.101–27.017)	0.03^*^
Barq	1.527 (0.367–6.354)	0.5	2.143 (0.491–9.351)	0.3	5.727 (1.022–32.103)	0.04^*^
Bisha	3.60 (0.491–26.398)	0.2	5.833 (0.525–64.823)	0.1	4.500 (0.570–35.519)	0.1
Khamis Mushayt	1.60 (0.436–5.868)	0.4	1.912 (0.513–7.131)	0.3	6.000 (1.186–30.349)	0.03^*^
Mahayil	4.800 (0.397–58.013)	0.2	0.556 (0.065–4.775)	0.5	6.750 (0.640–71.174)	0.1
Sarat Ubaida	2.00 (0.312–12.840)	0.4	5.833 (0.525–64.823)	0.1	7.500 (0.921–61.047)	0.06
Tharban	7.80 (1.162–52.353)	0.03^*^	2.292 (0.441–11.917)	0.3	3.937 (0.627–24.731)	0.1
The Majardah	1.800 (0.318–10.201)	0.5	7.500 (0.692–81.248)	0.09	18.00 (2.037–159.090)	0.009^**^
**Non-Asir region**						
AL Madinah AL Munawwarah	4.20 (0.586–30.096)	0.1	1.667 (0.269–10.334)	0.5	2.250 (0.285–17.759)	0.4
Al-Qassim	1.680 (0.322–8.756)	0.5	0.833 (0.162–4.295)	0.8	4.500 (0.670–30.320)	0.1
Hail	1.500 (0.255–8.817)	0.6	2.917 (0.407–20.899)	0.2	5.625 (0.747–42.359)	0.09
Jazan	2.400 (0.444–12.980)	0.3	2.500 (0.428–14.607)	0.3	13.500 (1.802–101.125)	0.01^*^
Mecca	1.467 (0.335–6.430)	0.6	2.500 (0.525–11.894)	0.2	5.500 (0.939–32.205)	0.05^*^
Najran	1.200 (0.164–8.799)	0.8	4.167 (0.358–48.440)	0.2	0.900 (0.64–12.583)	0.9
Riyadh	3.273 (0.829–12.921)	0.09	16.250 (2.551–103.494)	0.003^**^	7.031 (1.343–36.820)	0.02^*^
Tabuk	4.000 (0.693–23.089)	0.1	10.000 (0.944–105.921)	0.05^*^	2.000 (0.290–13.814)	0.4
Taif	Reference					

^*^p < 0.05; ^**^p < 0.001—significant.

Regional comparisons are presented as exploratory analyses due to the uneven regional distribution of the sample, with a predominance of respondents from the Asir region. These findings should be interpreted cautiously and are intended to generate hypotheses rather than establish definitive regional differences.

The bivariate analysis assessed the association between demographic characteristics and participants' KAP toward the use of AI in pharmacy practice (Table 9). Gender did not show any significant association with knowledge (OR = 0.812, *p* = 0.3), attitude (OR = 1.192, *p* = 0.5), or perception (OR = 0.871, *p* = 0.5). Similarly, age and occupational categories were not significantly related to these domains (*p* > 0.05). Although higher educational qualifications, particularly among pharmacists with master's degrees, exhibited relatively higher odds of good knowledge (OR = 12.00, *p* = 0.07), these differences did not reach statistical significance.

Exploratory regional analyses indicated statistically significant associations in selected locations. Within the Asir region, participants from Abha (*p* = 0.03), Barq (*p* = 0.04), and Khamis Mushayt (*p* = 0.03) showed higher odds of positive perception toward AI. In addition, respondents from Tharban demonstrated higher odds of good knowledge (OR = 7.80, *p* = 0.03), while those from The Majardah showed the strongest association with positive perception (OR = 18.00, *p* = 0.009).

Among non-Asir regions, exploratory findings showed that participants from Riyadh exhibited notable associations with attitude (OR = 16.250, *p* = 0.003) and perception (OR = 7.031, *p* = 0.02), while associations with knowledge did not reach statistical significance (OR = 3.273, *p* = 0.09). Jazan and Mecca demonstrated significant associations with perception (*p* < 0.05), and participants from Tabuk showed a significant association with attitude (OR = 10.00, *p* = 0.05).

Overall, these exploratory regional findings suggest localized variation in AI-related knowledge, attitudes, and perceptions; however, due to the predominance of respondents from the Asir region (60.1% Asir, 39.9% other regions), these results should be interpreted as hypothesis-generating and not as evidence of national regional differences.

## 5 Discussion

Before interpreting regional differences in knowledge, attitudes, perceptions, and needs related to artificial intelligence, it is essential to acknowledge the uneven regional composition of the study sample. A majority of respondents (60.1%) were from the Asir region, reflecting the convenience-based recruitment strategy rather than proportional national representation. Consequently, all regional comparisons presented in this study should be interpreted as exploratory rather than definitive. These findings are not nationally representative and should be viewed as hypothesis-generating observations, potentially reflecting localized educational exposure, digital accessibility, or institutional emphasis rather than true regional disparities across Saudi Arabia.

The demographic composition of the participants indicates that the study largely involved young adults, particularly those aged between 18 and 39 years, with a predominance of females. This pattern is consistent with the general demographic structure of pharmacy programs, where younger and predominantly female students are commonly enrolled. The higher representation of pharmacy students compared to diploma and graduate-level participants suggests a greater interest or exposure to emerging technologies such as AI among undergraduates. The concentration of participants from the Asir region reflects both the accessibility of the study population and the regional emphasis on integrating digital learning tools within pharmacy education. The overrepresentation of third- and fourth-year students further implies that individuals in advanced academic stages may possess more awareness and engagement with AI-related concepts. These findings are consistent with those reported by Hasan et al. (8), who also observed strong engagement with AI-related topics among senior pharmacy students.

Although a high proportion of participants (82.6%) reported awareness of artificial intelligence tools, fewer than half (42.3%) actively used them for academic or professional purposes, indicating a significant awareness–practice gap (*p* < 0.05). This finding suggests that awareness alone is insufficient to drive adoption in pharmacy education. Limited curricular integration of AI tools, concerns regarding data accuracy and the potential for false information, and ethical apprehensions related to academic misconduct may discourage routine use. In addition, the absence of institutional guidelines and formal training on responsible AI use may create uncertainty regarding acceptable academic and clinical applications. Similar awareness – use disparities have been reported in healthcare education, emphasizing that effective AI adoption requires structured curricular integration, faculty guidance, and competency-based training rather than passive exposure alone.

According to the current study, pharmacy students are well aware of AI. Our results are consistent with recent research that found high awareness of AI (8, 17). However, although most participants reported general awareness, deeper conceptual understanding remained limited, indicating a gap between familiarity and actual knowledge. This clarifies the paradox in which participants “knew of AI” but demonstrated low application scores. In our sample, low application scores and a lack of structured classroom instruction appear to be the main contributors to pharmacy students' limited understanding of AI. Additionally, pharmacy graduates generally reported lower AI-related knowledge compared with students, suggesting that exposure during training may not be consistently reinforced once graduates transition into practice. Some research found that university students have a moderate level of AI expertise (8). A Nigerian study found good knowledge of AI among most pharmacy students (18). Our findings also align with a study reporting inadequate AI knowledge among radiology students (5, 6), underscoring that surface-level awareness does not necessarily translate into functional competence across health disciplines. Another study which was done in Syria observed that 70% of students had a basic concept of AI. Overall, our participants demonstrated adequate awareness of AI at a basic level, but more advanced and practice-oriented knowledge remained suboptimal.

According to the KAP questionnaire, participants showed higher knowledge of AI terminology than of its practical advantages. This suggests that participants may be more familiar with the terminology and concepts related to AI than with its possible advantages and disadvantages. This discrepancy highlights a conceptual-vs.-functional knowledge gap, commonly seen in early-stage AI adoption environments. This disparity may result from instructors and students in pharmacy not receiving formal AI education and training, which is in line with other studies (17). Therefore, the results emphasize the necessity of adding more instruction and training on AI-related courses to pharmacy curriculum in order to enhance students understanding and proficiency in this field. Embedding AI into pharmacy education with clear learning outcomes, case-based applications, and assessment strategies could help translate conceptual awareness into practical competency. This could result in a more practical implementation of AI in pharmacy practice, paying special attention to fundamental computational concepts and AI terminology.

Importantly, the questionnaire demonstrated acceptable to high internal consistency across its domains, as indicated by the Cronbach's alpha values (Table 8), further supporting the robustness and reliability of the KAP findings presented in this study. Cronbach's alpha was used to assess the internal consistency of each scale, with values ranging from acceptable to excellent, indicating that the items within each domain consistently measured their intended constructs. Additionally, we discovered that although pharmacy students and graduates have a positive attitude toward AI (Table 3), worries about how AI may affect patient safety and job security persist. Pharmacists, medical professionals, and AI technology specialists should work together, communicate openly, and provide education to allay these worries. 76.6% of healthcare students and graduates had a positive and promising attitude toward AI in the clinical profession and its use in the future, according to a systematic review that looked at the attitudes, knowledge, and skills of healthcare students in the field (19). However, 23.4% of the students had a negative attitude toward AI and saw it as a threat to healthcare fields.

The pharmacy students and bachelor pharmacists have mixed perceptions of AI programs. Only a small proportion (12%−13%) considered AI-generated drug data accurate, while 14%−20% viewed it as potentially unreliable, and around one-third believed AI data might contain false information (*p* > 0.05). Despite these concerns, most participants recognized AI educational value, with 38%−42% agreeing it enhances learning, understanding, and motivation (*p* > 0.05). However, about a quarter expressed concerns that AI could enable academic dishonesty. Overall, respondents perceive AI as a helpful educational tool, but caution remains regarding data accuracy and ethical use. These perceptions need to be interpreted in the context of the specific AI tools that students and graduates currently use.

In interpreting the findings across Tables 3 and 5, it is important to distinguish between conceptual awareness of AI tools and actual hands-on use. The specialized AI systems listed in Table 3, such as IBM Watson, Path AI, and Zebra Medical Vision, are advanced platforms that typically require institutional licensing, clinical datasets, or integration into healthcare systems. These tools are not routinely accessible to students or early-career practitioners in academic settings. Consequently, participants may recognize these systems from coursework, scientific literature, or general exposure to AI in healthcare but have never used them in practice. In contrast, the tools reported in Table 5, such as ChatGPT-like large language models and voice-assistant technologies (e.g., Siri), are freely available, easy to access, and widely used for everyday academic tasks. This explains why conceptual recognition of specialized tools is relatively high, while actual usage is concentrated in general-purpose AI applications. This contrast further reinforces the awareness–practice gap observed in the study, whereby knowledge of AI does not translate into direct experience due to accessibility, training, and institutional resource limitations.

Unlike several earlier studies that focused primarily on traditional AI applications (e.g., rule-based decision support systems, robotics, or standalone diagnostic algorithms), our instrument intentionally included AI tools that pharmacy learners and graduates are currently most likely to encounter in their daily academic and professional work. These included large language models (such as ChatGPT-like platforms), AI-enabled drug-information resources, electronic prescribing and medication-management systems, and other data-driven clinical support tools. By focusing on this contemporary toolset, our findings complement existing literature and provide a more up-to-date picture of how AI is actually being used in pharmacy education and practice in Saudi Arabia, rather than replicating earlier tool lists that may no longer reflect current usage patterns. This also helps to explain why the tools reported in our results differ from those in some previous studies, which were conducted before the widespread adoption of generative AI.

There is a need to include AI education and training in pharmacy curriculum, since the current study also found that pharmacy graduates and students have little experience utilizing AI technologies in their practice. This finding highlights the paradox where students express positive attitudes but still lack practical exposure indicating that attitude alone does not translate into behavior without structured training. This result is in line with other research that found a disconnect between the promise of AI in healthcare and its actual application in clinical settings because of healthcare practitioner insufficient knowledge and expertise. These findings suggest that AI will likely be utilized more frequently in pharmaceutical and medical settings in the future (19). As a result, it is crucial that pharmacy students get the information and abilities required to use AI in everyday clinical settings. Many of the participants were using AI programs, with a high proportion reporting the use of ChatGPT or similar LLM-based tools (81.1%) and using AI for studying (83.5%) and for research-related tasks (67.0%). These findings were found to be similar with Robledo et al. study (10) and Kalaimani et al. (11) study which stated as participants practices are below half among total study population. Taken together, our results suggest that while AI-based tools are already being used informally for learning and research, their integration into structured clinical decision-making and supervised educational activities remains limited.

The bivariate analysis revealed no significant association between demographic factors (sex, age, occupation) and knowledge, attitude, or perception toward AI, indicating that awareness is relatively uniform across these groups. Participants with a master's degree showed higher odds of good knowledge, reflecting possible exposure to AI concepts in advanced pharmacy curricula. Regionally, respondents from the Asir region, particularly Abha, Barq, Khamis Mushayt, and Tharban, exhibited significantly higher positive perceptions, suggesting a growing technological receptivity. The Majardah showed the highest perception scores, implying strong regional engagement with digital innovation. This may be attributed to improved digital literacy and government-led health technology initiatives in the area. This regional variation may reflect differential access to technological resources and regional academic priorities rather than inherent differences in student capability. Non-Asir regions such as Riyadh, Mecca, Jazan, and Tabuk also displayed positive associations, with Riyadh showing significance across all three domains, underscoring its leadership in AI-driven healthcare. However, given the uneven regional distribution in our sample, this subgroup findings should be interpreted as exploratory rather than definitive. The overall findings indicate widespread but regionally varied AI awareness, with Asir showing notable advancement in perception. These results emphasize the need for continuous regional training and curriculum integration to enhance AI competence across pharmacy sectors in Saudi Arabia.

In comparing our findings with previous research from Jordan (3, 17), the Philippines (5, 10), and Syria (16), it is evident that pharmacy students across different countries demonstrate varying levels of awareness and acceptance of AI. While earlier studies reported mixed perceptions of AI in healthcare, our participants generally showed moderate to positive levels of awareness and attitudes. However, unlike some international findings where students expressed strong fears of AI replacing pharmacists, our study did not present evidence of such concerns. Instead, the attitudes observed in our sample remained balanced, reflecting both enthusiasm for AI integration and caution regarding its limitations. To maintain consistency with our reported results, we have removed unsupported comparisons and ensured that the interpretations align only with the findings presented in this study. To maintain internal consistency, we have focused our comparative interpretation on domains that were directly measured in this study and avoided extrapolating to constructs that were not assessed.

There was a clear interest among pharmacy students and bachelor pharmacists in integrating AI into both education and practice. A substantial proportion expressed the need to include AI topics in the pharmacy curriculum and to implement AI-enabled tools in hospitals and community pharmacies, highlighting the perceived potential of AI to enhance patient care. Additionally, many participants showed a strong desire to gain further knowledge about AI applications in healthcare settings.

Interestingly, our findings indicated that attitudes toward AI and knowledge of AI were positively correlated. This reinforces the behavioral theory that increased knowledge promotes more favorable perceptions, supporting targeted AI educational strategies. This suggests that raising faculty and student understanding of AI might result in a greater acceptance and use of AI techniques in pharmacy practice. Furthermore, our findings showed a strong positive relationship between general KAP scores and AI knowledge, indicating that knowledge is a major factor in influencing behavior change toward the use of AI in pharmacy practice. These findings suggest that pharmacists may view AI as a collaborator in enhancing patient care and advancing pharmacy practice (20).

Our research has important practical implications for the use of AI in pharmacy, including the development and assessment of educational interventions aimed at improving pharmacist practical skills in this area. First, it highlights the importance of AI education in pharmacy curricula, with coursework incorporating references to AI literature, computational concepts, and AI terminology; second, it highlights the need to address concerns regarding job security and patient safety. Carefully designed AI curricula could also showcase real-world use cases, such as decision support, medication adherence monitoring, and AI-assisted drug development, to demonstrate how AI augments rather than replaces professional judgment.

Although all experimental groups were included to provide complete comparative insights, one group had a relatively small sample size. This limited number may reduce the statistical power and increase the risk of biased estimates when interpreting between-group differences. Therefore, findings involving this small group should be interpreted with caution. Future studies with larger and more balanced group sizes are recommended to validate these observations.

Despite efforts to design a culturally appropriate survey instrument, linguistic and cultural variations may have influenced how participants interpreted certain items. Further research is needed to better explore participants' deeper understanding of AI and to address the ethical and legal implications associated with its integration in healthcare. Future studies may overcome these limitations by adopting longitudinal designs and expanding the sample to include more regions across Saudi Arabia. Another limitation is the 1-year gap between data collection (November 2024) and manuscript submission. Given the rapid advancements in AI and its growing adoption in pharmacy practice and education, attitudes and knowledge may have evolved since data collection. Nevertheless, the present findings provide valuable baseline insights into early adoption trends within the country. Additionally, because AI technologies are rapidly evolving, periodic reassessment of pharmacy students' and graduates' attitudes will be essential to ensure that educational strategies remain relevant and aligned with emerging advancements.

### 5.1 Limitations

Several limitations should be considered when interpreting the findings of this study. First, the sample exhibited a notable regional imbalance due to non-probability, convenience-based online recruitment. Because invitations were distributed through open electronic channels without the ability to track how many individuals viewed the survey link, a conventional response rate could not be calculated. Approximately 60% of respondents were from the Asir region and 40% from other regions of Saudi Arabia. This uneven representation limits the generalisability of the findings to the national level and underscores that the regional comparisons reported in this study should be interpreted as exploratory rather than definitive.

Second, the reliance on self-reported questionnaire data introduces the possibility of response bias and social desirability bias. The study population was also limited to pharmacy students and graduates, excluding other healthcare professionals whose perspectives may differ, thereby constraining external validity. In addition, the cross-sectional design precludes causal inference and does not permit assessment of changes in knowledge, attitudes, or practice over time.

Third, the temporal gap between data collection (November 2024) and manuscript revision represents an important limitation. Given the rapid evolution of AI technologies and their expanding integration into pharmacy education and practice, participants' knowledge, attitudes, and usage patterns may have evolved since the time of data collection. Accordingly, the present findings should be interpreted as a baseline snapshot during an early phase of AI adoption, and future studies using longitudinal or repeated cross-sectional designs are recommended.

Fourth, a conceptual limitation was identified within the perception domain, which contained heterogeneous items assessing perceived data accuracy, educational value, and ethical concerns, including belief-based evaluations of AI accuracy rather than objectively verifiable knowledge items. This conceptual breadth likely contributed to the moderate internal consistency (Cronbach's α = 0.592). Future surveys should differentiate more clearly between belief-based perceptions, objective knowledge, and attitudinal constructs to enhance psychometric precision and interpretability.

Additionally, the practice domain did not impose a fixed recall window (e.g., past month or past year), which may have resulted in variability in how participants interpreted “typical” AI use. Similarly, some contingency table cells contained small counts, which may affect the appropriateness of chi-square testing for those specific comparisons and should be interpreted with caution. In addition, some subgroup comparisons involved relatively small participant numbers, which may reduce statistical power and increase the risk of unstable estimates. Findings from these smaller subgroups should therefore be interpreted with caution Finally, given the number of statistical tests performed, the possibility of Type I error inflation cannot be ruled out. Future research with larger samples and, where appropriate, statistical correction procedures (such as Bonferroni adjustments) is warranted.

### 5.2 Implications of AI for pharmacy education and practice

#### 5.2.1 Understanding the awareness-practice gap

The study revealed a substantial discrepancy between participants' high awareness of AI tools (82.6%) and their comparatively low level of practical utilization (42.3%), indicating a clear awareness-practice gap with important educational implications. Several factors appear to contribute to this divide. First, despite widespread familiarity with AI concepts, pharmacy programs have not yet integrated structured AI training into coursework, leaving students with conceptual awareness but limited opportunities to apply AI tools in academic or clinical tasks. This curricular absence is likely compounded by the limited faculty-led incorporation of AI-supported learning activities, as suggested by the mismatch between positive attitudes toward AI and low levels of practical engagement. Without guided exposure, students may lack the confidence and competence needed to translate awareness into routine use.

Second, the gap is reinforced by participants' concerns regarding the reliability and accuracy of AI-generated information. Approximately one-third expressed apprehension that AI tools may produce false or misleading outputs, highlighting uncertainty about when and how AI can be trusted. Such concerns may discourage students from using AI in medication-related or decision-support contexts without explicit training on verification and critical appraisal. Additionally, ethical and academic integrity considerations, reflected in the 27.9% who feared potential misuse or academic dishonesty, may further inhibit adoption in formal learning environments. Finally, regional disparities in AI engagement indicate that access to digital infrastructure, institutional readiness, and exposure to innovative teaching strategies vary across the country, potentially magnifying the awareness–practice gap in regions with less developed AI-supportive environments.

Collectively, these findings suggest that awareness alone is insufficient for meaningful adoption. Closing the awareness-practice gap requires structural, curricular, and institutional interventions that build competence, trust, and responsible use patterns among pharmacy students and graduates.

#### 5.2.2 Implications for pharmacy education and practice

The findings of this study highlight several actionable strategies for strengthening pharmacy education and practice in response to emerging AI technologies. The pronounced awareness–practice gap, combined with participants' concerns about accuracy, ethical use, and uneven regional engagement, underscores the need for a coordinated and structured educational approach. To address this, pharmacy programs should consider integrating a dedicated AI module of 1–2 credit hours during early professional training and reinforcing it in clinical courses. Core instructional elements should include: (i) foundational principles of AI and machine learning in healthcare; (ii) applications of AI in drug information retrieval, pharmacovigilance, and clinical decision support; (iii) limitations, uncertainty, and potential biases in AI systems; and (iv) ethical, legal, and data-governance considerations relevant to patient care (21). Assessments should move beyond knowledge recall to include case-based assignments, structured critical appraisal of AI-generated outputs, and OSCEs incorporating AI-assisted scenarios, thereby ensuring students can translate conceptual understanding into evidence-based and responsible practice.

The limited practical use of AI tools despite generally positive attitudes suggests insufficient faculty-led integration of AI-enhanced learning. The gap between high awareness and positive attitudes on one hand, and low practical use on the other, indicates that structured faculty engagement with AI technologies is currently limited. Faculty development initiatives are therefore essential and should emphasize foundational AI literacy, hands-on experience with educational AI platforms, and training in supervising and assessing responsible student use. Workshops, short certification programs, or continuing professional development (CPD) modules can equip educators with the competencies needed to integrate AI activities into teaching while maintaining academic rigor (22, 23).

Concerns about data accuracy were reported by approximately one-third of participants, reflecting uncertainty about the reliability of AI outputs. Embedding training on the critical appraisal of AI-generated information is therefore vital. Curriculum strategies should include instruction on validating AI outputs against authoritative drug information sources, identifying algorithmic bias, scrutinizing source credibility, and triangulating AI-generated recommendations with clinical guidelines and primary literature (24). Strengthening students' ability to evaluate and contextualize AI information will mitigate the risks associated with uncritical reliance on digital tools.

Additionally, exploratory regional findings revealed higher engagement in selected Asir localities alongside lower engagement in several non-Asir regions. These disparities indicate a need for region-sensitive capacity-building strategies to ensure equitable development of AI competencies nationwide. National pharmacy education bodies could support this through shared digital learning platforms, inter-institutional collaboration, and centralized AI training resources that reduce regional differences in access to AI education and digital infrastructure.

Finally, concerns related to academic dishonesty, reported by 27.9% of participants, underscore the need for clear, institution-wide policies guiding the ethical use of AI. Pharmacy schools should establish explicit guidelines delineating acceptable and unacceptable AI-assisted academic practices, including disclosure requirements, limitations on AI use during assessments, and standards for AI use in clinical decision-making (23, 25). Aligning these policies with national regulatory frameworks and professional expectations will promote responsible AI integration while preserving academic integrity and patient safety.

Together, these recommendations demonstrate that effective AI integration in pharmacy education requires coordinated curricular innovation, well-prepared faculty, systematic ethical oversight, and regionally inclusive implementation strategies. Addressing these areas concurrently may help bridge the awareness–practice gap identified in this study and better prepare future pharmacists for safe, competent, and evidence-based use of AI technologies in patient care.

## 6 Conclusion

This study provides insight into the knowledge, attitudes, and practices related to artificial intelligence among pharmacy students and graduates drawn from a multi-regional convenience sample predominantly representing the Asir region of Saudi Arabia. Participants demonstrated high awareness of AI and generally positive attitudes toward its integration into pharmacy education and practice; however, practical utilization remained limited, indicating a persistent awareness–practice gap.

Although exploratory regional analyses suggested variability in AI perceptions across locations, these findings should be interpreted cautiously and are not nationally representative due to the uneven regional distribution of the sample. The findings reflect strong AI readiness within the surveyed cohort, but the regional imbalance limits generalizability to the national level.

Collectively, the results underscore the importance of systematically integrating AI education into pharmacy curricula, while simultaneously addressing students' perceptions, concerns, and ethical understanding of AI tools, through structured training, ethical guidance, and supervised application. Future studies using stratified or probability-based sampling frameworks are essential to validate regional patterns, enhance national generalizability, and guide evidence-based policy and curriculum development across Saudi Arabia.

## Data Availability

The original contributions presented in the study are included in the article/supplementary material, further inquiries can be directed to the corresponding author.
